# Print versus a culturally-relevant Facebook and text message delivered intervention to promote physical activity in African American women: a randomized pilot trial

**DOI:** 10.1186/s12905-015-0186-1

**Published:** 2015-03-27

**Authors:** Rodney P Joseph, Colleen Keller, Marc A Adams, Barbara E Ainsworth

**Affiliations:** Arizona State University, College of Nursing and Health Innovation, 500 N. 3rd Street, Phoenix, AZ 85004 USA

## Abstract

**Background:**

African American women report insufficient physical activity and are disproportionally burdened by associated disease conditions; indicating the need for innovative approaches to promote physical activity in this underserved population. Social media platforms (i.e. Facebook) and text messaging represent potential mediums to promote physical activity. This paper reports the results of a randomized pilot trial evaluating a theory-based (Social Cognitive Theory) multi-component intervention using Facebook and text-messages to promote physical activity among African American women.

**Methods:**

Participants (N = 29) were randomly assigned to receive one of two multi-component physical activity interventions over 8 weeks: a culturally-relevant, Social Cognitive Theory-based, intervention delivered by Facebook and text message (FI) (n = 14), or a non-culturally tailored print-based intervention (PI) (n = 15) consisting of promotion brochures mailed to their home. The primary outcome of physical activity was assessed by ActiGraph GT3X+ accelerometers. Secondary outcomes included self-reported physical activity, physical activity-related psychosocial variables, and participant satisfaction.

**Results:**

All randomized participants (N = 29) completed the study. Accelerometer measured physical activity showed that FI participants decreased sedentary time (FI = −74 minutes/week vs. PI = +118 minute/week) and increased light intensity (FI = +95 minutes/week vs. PI = +59 minutes/week) and moderate-lifestyle intensity physical activity (FI = + 27 minutes/week vs. PI = −34 minutes/week) in comparison to PI participants (all *P’s* < .05). No between group differences for accelerometer measured moderate-to-vigorous intensity physical activity were observed (*P* > .05). Results of secondary outcomes showed that in comparison to the PI, FI participants self-reported greater increases in moderate-to-vigorous physical activity (FI = +62 minutes/week vs. PI = +6 minutes/week; *P* = .015) and had greater enhancements in self-regulation for physical activity (*P* < .001) and social support from family for physical activity (*P* = .044). Satisfaction with the FI was also high: 100% reported physical activity-related knowledge gains and 100% would recommend the program to a friend.

**Conclusions:**

A culturally-relevant Facebook and text message delivered physical activity program was associated with several positive outcomes, including decreased sedentary behavior, increased light- and moderate-lifestyle intensity physical activity, enhanced psychosocial outcomes, and high participant satisfaction. Future studies with larger samples are warranted to further explore the efficacy of technology-based approaches to promote physical activity among African American women.

**Trial Registration:**

ClinicalTrials.gov NCT02372565. Registered 25 February 2015.

## Background

African American women are one of the least physically active demographic groups in the United States. Only 36% meet the national recommendation of 150 minutes per week of moderate-to-vigorous physical activity [1]. Low physical activity levels are especially worrisome because African American women are disproportionally burdened with medical conditions associated with inactivity, including colon and breast cancers, overweight/obesity, type II diabetes, and cardiovascular disease [2]. Such disparities require innovative approaches to promote physical activity in this high-risk, underserved population.

Technology-based platforms (i.e. Internet, mobile phone, social media, text message, etc.) are promising eHealth and mHealth options to help decrease physical activity-related health disparities among African American women. Nationally representative data show that African American adults access the Internet at rates similar to Non-Hispanic Whites [3]. African American adults are more likely to own a mobile phone (87% of African American versus 80% of Whites) [4] and have the highest prevalence of smartphone ownership when compared to other race/ethnic groups [3]. Moreover, African Americans are more likely to use their mobile phones to access social media websites (i.e. Facebook, Twitter, etc.) [5] and send and receive text messages [6]. Despite the high prevalence of Internet, social media, and text message use among African American women, few interventions have used such technologies to deliver culturally relevant physical activity interventions to African American women. Our review of the literature found no studies using social media to promote physical activity exclusively among African American women and only six studies using either a website [7-9] or text message-based approach [10-12] to promote physical activity in African American women. The lack of culturally-relevant technology-based approaches to promote physical activity among African American women represents a missed opportunity given the high levels of technology use in this population and substantial evidence suggesting that technology-based physical activity promotion efforts are effective [13-17].

Existing and commonly used social media and text messaging infrastructures may be especially advantageous for delivering physical activity promotion interventions to African American women. Combined social media and text messaging interventions have been used to successfully promote various health behaviors (i.e., physical activity, dietary behaviors) [18,19] and weight loss [20,21] in previous research and have several advantages compared to traditional face-to-face or mailed print-based physical activity interventions. Advantages include lower participant burden associated with not having to attend in-person intervention sessions and 24-hour access to intervention materials. Moreover, from a public health standpoint, technology-based platforms can provide researchers the ability to reach a large number of people at a relatively low cost; which can ultimately lead to a greater public health impact of physical activity promotion efforts.

The purpose of the current randomized pilot trial was to evaluate a multi-component, culturally-relevant intervention delivered using Facebook and text-messaging designed to promote physical activity among African American women. The Facebook and text message intervention was based on tenets of Social Cognitive Theory [22], a behavioral health theory that explains human behavior in a triadic and reciprocal model where one’s personal, environmental, and behavioral factors all interact to influence behavior. The Social Cognitive Theory is one of the most frequently used behavioral theories in physical activity research [13,23] and was selected as the theoretical background in the current study due to its particular focus on the interaction between one’s personal characteristics and his/her social environment: as these are two key factors associated with physical activity behavior in African American women [24]. Similarly, Facebook, the largest and population social media site in the U.S. [5], and text messaging were selected as the primary delivery channels for the intervention materials due their popularity and high use among African American women. Given the paucity of published reports using a theory-based technology intervention to promote physical activity among African American women, this study provides important insight regarding the acceptability and feasibility of using social media and text message technologies to promote physical activity in African American women.

## Methods

### Study design

An 8-week, 2-arm randomized pilot trial was implemented. Sample size was determined by financial feasibility. Twenty-nine African American women were assigned by the first author using a random number allocation sequence (SPSS, version 21) to receive either a Social Cognitive Theory-based, culturally-relevant physical activity intervention delivered via Facebook and text messaging (hereafter referred to as FI) or to a standard print-based intervention (PI), consisting of non-culturally tailored physical activity promotion brochures developed by the American Heart Association, mailed to their home address. Participants also wore an Omron HJ-720IT pedometer and kept a calendar to track and self-monitor their daily step counts. The Omron HJ-720IT pedometer is a validated [25] dual-axis acceleration sensor that can be worn (vertically or horizontally) anywhere on the body (i.e., hip, pocket, purse, etc.). The pedometer provides an estimate of: a) total of steps per day, and b) minutes per day of walking at a cadence associated with moderate-intensity physical activity (i.e., >100 steps per minute). This pedometer was selected because it allowed participants to evaluate both their daily step counts and minutes per day of moderate-intensity physical activity so they could compare their activity to the national physical activity guidelines.

### Participants

Participants were recruited in February 2014 from the metropolitan area of Phoenix, Arizona. Recruitment strategies included email distribution lists (ex., African American sororities, churches), fliers posted at local businesses, advertisements placed in a local African American newspaper, and postings on a local social event website for African Americans. Facebook advertisements were not used to recruit participants due to both financial restraints and the limited number of participants that could be enrolled in the study (i.e., Facebook advertisements are fee-based and have high reach potential that may have resulted in many more women wishing to participate in the study than financial feasible). Women were eligible for the study if they: a) self-identified as African American and female, b) were insufficiently active (<150 min/week of moderate-intensity physical activity as assessed by the Short Version of the International Physical Activity Questionnaire), c) were aged 24–49 years, d) had an active Facebook account at the time of eligibility screening, and e) could to read and write in English. Only adult women aged 24–49 were recruited because the intervention was tailored to adult-aged African American women of childbearing age and because national data show that social media and text message use declines after the age of 50 [4,5]. Exclusion criteria included: a) concurrent participation in another physical activity promotion program (research or commercial), b) pregnancy or planned to become pregnant in the next 6 months, and c) a contraindication to exercise as indicated by the Physical Activity Readiness Questionnaire (PAR-Q) unless written permission was provided for the participant’s primary care physician.

### Procedures

The study was approved by the Arizona State University Institutional Review Board. After reading or hearing about the study from recruitment materials, African American women interested in participation contacted study staff via email or telephone. Potential participants were screened for eligibility by telephone using an eligibility screener developed for the current study and the Physical Activity Readiness Questionnaire. Eligible participants were scheduled for their baseline study visit.

At the baseline study assessment, participants provided informed consent, completed demographic, psychosocial and behavioral questionnaires, and had their height and weight assessed by trained study staff. Participants were then provided an accelerometer and instructed to wear it during all waking hours for the next 7 days. At the end of the 7-day wear period, participants returned their accelerometer to study staff and received $10 for participation. Approximately one week following the accelerometer return, participants were notified via telephone of their group assignment and scheduled for a study orientation visit that would take place the subsequent week. Separate group orientations were held for each study arm as participants were blinded to the differences in physical activity promotion materials between arms (i.e., culturally relevant vs. non-culturally relevant). Participants who were unable to attend their group orientation visit were provided a one-on-one orientation by a trained staff member. The purpose of the orientation session was to give participants a brief overview of the study group they were assigned, provide instructions on how to properly wear the pedometer, and to verify they that the research team had correct contact information on file. The orientation session was also when participants randomized to the FI were added to the study Facebook group.

Follow-up data collection procedures were similar to baseline procedures, with anthropometric, psychosocial, treatment acceptance, and physical activity data collected approximately one week after the end of the intervention period. Upon return of the accelerometer after the 7-day wear period, participants were provided an additional $15 for study participation. The last participant completed the intervention in June 2014.

### Physical activity interventions

#### Facebook and text message intervention (FI)

The FI was comprised four primary intervention components designed to increase moderate-intensity physical activity among participants to the nationally recommended levels of 150 minutes per week [26]. These four components targeted one or more of the constructs of the Social Cognitive Theory and included: 1) Weekly physical activity promotion materials posted on the group Facebook wall, 2) Discussion topics and participant engagement on the group Facebook wall, 3) Motivational text messages promoting physical activity, and 4) Adaptive pedometer-based self-monitoring and goal-setting program. Email was also used as a communication channel between study staff and participants (see Adaptive pedometer-based self-monitoring and goal setting program below).

##### Weekly Facebook materials

Participants randomized to the FI received a Social Cognitive Theory-based, culturally-relevant physical activity promotion intervention delivered through text messages and the social media website Facebook. These culturally relevant-intervention materials were developed specifically for the current study and were informed by the first author’s previous research with African American women [8,9,27], prominent determinants of physical activity among African American women [24,28], and published literature focused on culturally-tailoring lifestyle interventions to minority populations [29-31]. Specifically, intervention materials: (a) highlighted the low physical activity levels of African American women and associated disease burden (e.g., obesity prevalence and increased risk for chronic disease conditions, including colon cancer, cardiovascular disease, and type II diabetes) [2], (b) addressed prevalent sociocultural norms and barriers to physical activity commonly reported among African American women (e.g., familial and caretaking roles of African American women, lack of social support, hair concerns, and body shape preferences [24]), and (c) included images of African American women with varying body sizes and skin tones engaging in physical activity.

The Facebook group was “closed”, allowing only participants enrolled in the study to view the information posted on the Facebook group wall and view the profiles of other participants who are members of the group. The theory-based, physical activity promotion materials were delivered weekly (every Monday) on the study’s Facebook group wall. These materials were electronic print/image-based materials written at the 10th grade reading level or lower and followed a sequential pattern of physical activity-related topics with the overarching goal of increasing physical activity. Specific behavioral components of the Social Cognitive Theory that underpinned the intervention are described in Table 1. Weeks 1–6 focused on physical activity adoption by presenting new information and Social Cognitive Theory concepts to study participants each week. Weeks 7–8 reviewed and reinforced information presented in the first 6 weeks and focused on maintenance of increased physical activity after the conclusion of the intervention period.

**Table 1:** **Weekly physical activity topics and materials delivered using Facebook and targeted Social Cognitive Theory constructs**

Week	Physical activity topic	Group discussion question	Social Cognitive Theory constructs targeted
1	Overview of the national physical activity recommendations, health benefits of physical activity, and physical activity statistics among African American women.	What are your thoughts on the physical activity statistics among African American women statistics? How can you incorporate more physical activity into your daily routine?	• Behavioral Capability
			• Self-regulation
2	Developing a physical activity plan that works for you.	What types of physical activity do you enjoy doing? What tips can you provide the group to help increase daily physical activity?	• Behavioral Capability
3	Barriers to physical activity among African American women and strategies to overcoming barriers.	What are your specific barriers to physical activity and what the strategies you will use to help overcome them?	• Behavioral Capability
			• Self-efficacy
4	Developing a social support network to promote physical activity.	Who are potential sources of social support for physical activity?	• Social Support
5	Strategies for incorporating short bouts of physical activity into your daily routine to achieve national physical activity recommendations.	What strategies can you use each day to engage in more physical activity?	• Self-regulation
6	Testimonials from African American women on how they successfully incorporate physical activity into their daily lives.	If you were to give a testimonial to other participants on how you maintain a physically active lifestyle, how would it sound?	• Outcome Expectations
7	*Revisited:* National physical activity recommendations, Barriers to physical activity among African American women and strategies to overcoming barriers, and strategies to incorporate more physical activity into your life.	How have you overcome the barriers to physical activity you had prior to starting the study?	• Behavioral Capability
			• Self-regulation
			• Self-efficacy
8	Strategies for maintaining a physical active lifestyle after the intervention.	How will you continue to be physically active?	• Outcome expectations

##### Weekly Facebook discussion topics

Weekly physical activity promotion topics were accompanied by discussion prompts (e.g., “What strategies can you use each day to engage in more physical activity?”) to encourage participant dialogue on the group Facebook wall (see Table 1). Discussion prompts were posted every Wednesday to the Facebook wall to allow participants time to read and reflect on the week’s preceding physical activity promotion posts. The purpose of the weekly discussion topics was for participants to share their personal experiences with physical activity and to give/receive social support for physical activity. Study staff did not engage in dialogue with participants on the group wall beyond posting the weekly discussion topics, as this component of the program was designed to be participant driven. The only exception was one occasion when study staff intervened to correct inaccurate physical activity information posted by a participant.

##### Motivational text messages

FI participants received three text messages to motivate and promote physical activity each week. These text messages acted as another mechanism of social support and provided: (a) tips on strategies to increase physical activity throughout the day (e.g., “Set aside time today for several 10–15 minute walks. Walking 30 minutes at a moderate-intensity on 5 days each week = 150 minutes!”), (b) information on how to overcome barriers to physical activity (e.g., “Don’t let the lack of child care interfere with your physical activity routine. Take a walk with your whole family this weekend”.), (c) reminders of the health benefits of physical activity (e.g., “Physical activity promotes bone health and reduces the risk of bone fractures and osteoporosis”.), and (d) motivational/inspirational tips and quotes to participants (e.g., “ ‘Each person must live their life as a model to others’. - Rosa Parks”). The text message library consisted of 24 total messages (i.e., 3 text messages per week for 8 weeks) and all participants received the exact same text messages over the duration of the study. Additionally, the first text message delivered each week also informed participants that a new physical activity promotion topic of the week was posted on the group Facebook page. The content of the text messages and frequency of delivery (i.e., 3 messages per week) were derived from our previous work that showed African American women preferred to receive a variety of physical activity-related text messages and that receiving 3 text messages per week was ideal while enrolled in a physical activity promotion program [12].

##### Adaptive pedometer-based self-monitoring and goal-setting program

FI participants received weekly individualized step goals and social reinforcement from study staff via email. This component of the study was designed to target the Social Cognitive Theory constructs of self-regulation, reinforcement, and self-efficacy. Individualized step goals were based on an adaptive goal-setting and feedback approach adapted from previous research [32]. As opposed to a static step goal approach (i.e., goal of 10,000 steps per day), the adaptive approach assumed within-person variability and used this variance to adjust step goals each week to ultimately increase daily physical activity over time. Specifically, the adaptive goal-setting and feedback algorithm was based on a rank-order percentile algorithm. This percentile algorithm required: 1) continuous and repeated measurements of physical activity (i.e., steps per day), 2) ranking steps per day from lowest to highest, and 3) calculation of a new weekly step goal based on a 60th percentile criterion. For example, if a participant reported the following daily steps during a week of the study (ranked from highest to lowest): 2500, 3500, 4200, 4500, 5100, 5500, 6000, her step goal for the next week (i.e., next 7 days) of the study would be 5100 (based on the 60th percentile of the previous week’s steps). We selected to use an adaptive approach with the FI group because it allowed for study staff to individually tailor step count goals to participants. Rather than asking all participants to immediately increase their step counts to a static goal of 8,000-10,000 steps per day during the first week of the study (as is frequently done in physical activity promotion research), the adaptive approach provided participants with more realistic step goals to achieve because they were based on their performance. This allowed study staff to slowly increase step counts each week (given participants were able to achieve their step goals) with the ultimate goal of increasing daily steps the general public health goal of 8,000-10,000 steps per day.

Participants emailed or texted study staff their daily step counts from the previous week on Sunday of each week. While the method of reporting weekly step counts to study staff was based on participant preference, almost all of the participants reported by email (only one participant regularly reported her weekly step counts to study staff via text message). Study staff then calculated each participant’s new step goal for the upcoming week and provided them brief standardized feedback and reinforcement via email on their progress (e.g., “Last week you achieved your step goal on 4 days —let’s see if you can meet your step goal on all 7 days this week. Your new minimum step count goal for the week is 8000 steps per day” or “Last week you achieved your step goal on all 7 days. Congratulations! Keep up the hard work and see if you can continue to meet your goal again on all 7 days this week. Your new minimum step count goal for the week is 9,000 steps per day).

#### Print-based Intervention (PI)

##### Mailed print-based component

Participants randomized to the PI served as the comparison group of the study and were mailed four non-culturally tailored, self-help booklets promoting physical activity and health produced by the American Heart Association [33-36]. Booklets were mailed to their home address every two weeks over the 8 week study (i.e., weeks 1, 3, 5, 7). These high quality booklets provided general information on risk factors for cardiovascular disease, the benefits of physical activity, tips and strategies to increase daily physical activity, and encouraged participants to perform a minimum of 150 minutes of MVPA per week. Similar versions of these booklets have shown efficacious in promoting physical activity in mostly White female populations [37,38].

##### Static pedometer-based self-monitoring program

Participants randomized to the PI were instructed to achieve a static goal of 8,000-10,000 steps each day. In order to attention-match for interactions with study staff in the FI, participants in this group also emailed their daily step counts on weekly basis to study staff. Study staff responded to the email using brief standardized feedback and reinforcement to encourage participants to continue to try and achieve 8,000-10,000 steps per day (e.g., “Last week you achieved your goal of 8,000-10,000 steps per day on 4 days. This week, see if you can meet your goal of 8000–10000 steps per day every day” or “Last week you achieved your goal of 8,000-10,000 steps on all 7 days”. This week see if you can keep your daily steps between 8,000 and 10,000. Keep up the great work!).

### Outcome measures

#### Physical activity

The ActiGraph GT3X+ accelerometer was the primary measure of physical activity in study. The GT3X+ assesses time spent in sedentary, light, moderate, and vigorous activity physical activity, as well as provides an estimate of daily step counts. Participants were instructed to wear the accelerometer on their right hip during waking hours for a consecutive 7-day period at both baseline and 8-weeks. Accelerometer data were downloaded and processed with ActiLife Software (version 6.10.0) using a 60-second epoch without the low frequency extension. To be considered as a valid assessment (and subsequently included in data analysis), participants were required to wear the accelerometer for at least 10 hours per day on at least four days during the seven day wear period. Non-wear time was defined as 60 minutes of consecutive zero counts with allowance of a 2 minute spike tolerance of less than 100 counts per minute. Cut-points developed from controlled laboratory experiments were used to estimate minutes of activity performed at various intensity levels: sedentary (0–99 counts/minute) [39], light (100–759 counts/minute), moderate-lifestyle (760–1951 counts/minute) [40], moderate (1952–5725 counts/minute), and vigorous (>5725 counts/minute) [41]. Additionally, accelerometer data were analyzed to provide an estimate of moderate-to-vigorous physical activity performed in bouts of ten minutes or greater (which coincides with the Physical Activity Guidelines for Americans: http://www.health.gov/paguidelines/). These ten-minute activity bouts were defined as achieving the aforementioned cut-point for moderate-intensity activities or greater (i.e., >1951 counts/minutes) for at least ten consecutive minutes, with allowance of one to two minutes below these thresholds during the ten minute period.

As a secondary physical activity measure, participants completed the 2-item Exercise Vital Sign questionnaire developed by Kaiser Permanente at Southern California [42]. This questionnaire asks participants to report their frequency (days per week) and duration (minutes per day) of moderate-to-vigorous intensity physical activity (e.g., like a brisk walk) during the past week. The questionnaire is scored by multiplying the days x minutes of physical activity performed to create an estimate of minutes per week of at least moderate-to-vigorous physical activity. The Exercise Vital Sign has been previously validated for accuracy against national population-based physical activity surveillance surveys (i.e., NHANES and the California BRFSS) [42].

### Social Cognitive Theory variables

Self-efficacy for physical activity was assessed by the Exercise Confidence Survey [43]. This 12-item survey has previously established reliability and validity [43] and demonstrated reliability estimates (Cronbach’s alpha) of 0.71 and 0.85 at baseline and 8 weeks respectively in the current study.

Social support for exercise was evaluated using the Social Support for Exercise Survey [44]. This scale provides two separate outcomes for social support: one in reference to family support (10-items) and the other in reference to support from friends (10-items). The Social Support for Exercise scale has previously established validity [44]. In the current study, reliability estimates for the family subscale were 0.93 and 0.90 at baseline and 8 weeks; estimates for the friends subscale were 0.79 and 0.92.

Self-regulation for physical activity was assessed by the Self-Regulation Scale from the Health Beliefs Survey [45,46]. This 10-item questionnaire has been previously validated [45,46] and has been used in previous research with African American women [8,9]. In the current study, the Self-Regulation Scale demonstrated reliability estimates (Cronbach’s alpha) of 0.57 and 0.65 at baseline and 8 weeks, respectively.

The Outcome Expectation Scale for Exercise (9-items) [47] was used to assess outcome expectations for physical activity. This scale has been validated in African American populations [48] and demonstrated reliability estimates (Cronbach’s alpha) of 0.82 and 0.83 in the current study.

### Body Mass Index (BMI)

A senior staff member measured participant’s height and weight at baseline and 8 weeks. To ensure consistency, the same staff member conducted measurements for all study participants. Weights were measured to the nearest kilogram using a Tanita TBF-300A digital scale. Height was measured to the nearest centimeter using a Seca 213 portable stadiometer. BMI was computed as weight in kilograms divided by height in meters squared.

### Feasibility and acceptability

Feasibility and acceptability of both interventions were assessed by satisfaction surveys adapted from previous research [8,49,50]. The FI satisfaction survey consisted of 26-items and evaluated participant acceptance regarding the content of the culturally-relevant physical activity promotion materials delivered via Facebook and text message, as well as the technology based platforms used to deliver the intervention materials. The PI satisfaction survey (19-items) included similar concepts as the FI survey as they related to the print-based physical activity promotion materials. Sample questions include: “Overall, how helpful do you find the [Facebook posts] or [print materials] for promoting physical activity?” and “Would you recommend the physical activity promotion program you received to a friend?”

### Fidelity of intervention delivery/participant engagement

Fidelity of intervention delivery was evaluated by several indices. Study staff kept log books to document delivery of intervention materials to participants. Participant receipt of the Facebook materials was evaluated by analytic tracking software provided by Facebook which recorded the names of participants that had viewed and “liked” the Facebook postings. Receipt of text-messages was evaluated by the number of messages that were returned as “undeliverable” by the mobile phone service (i.e. message failed to be delivered), and participants’ self-reported receipt of the text messages (assessed at the 8-week follow-up visit). Participant receipt of the print-based materials was determined by number of mailings that were “returned to sender” via the U.S. Postal Service and number of participants self-reporting receipt of print-based materials at the 8-week follow-up.

### Statistical analyses

Analyses were conducted with SPSS version 21. Descriptive statistics (i.e. means, medians, and percents) were calculated for all demographic, psychosocial, anthropometric, and behavioral outcomes. Mann–Whitney and chi-square tests were used to evaluate whether participant characteristics differed according to group assignment at baseline. Wilcoxon signed rank tests were used to examine within-group baseline to 8-week changes in physical activity, BMI, and Social Cognitive Theory construct outcomes. ANCOVAs (controlling for baseline values for each respective outcome) were conducted to examine between-group pre-post intervention differences for changes in outcomes. Effect sizes for ANCOVA analyses were hand calculated using the eta squared (*η*^*2*^) test statistic. Because distributions for two of the Social Cognitive Theory measures were skewed (i.e. social support from friends and social support from family), these variables were square root transformed prior to performing ANCOVA analysis. Additionally, accelerometer measured vigorous-intensity physical activity was not analyzed independently using ANCOVA because this variable could not be adequately transformed to meet assumptions necessary to perform ANCOVA (however, vigorous-intensity physical activity is included with moderate-intensity physical activity in the variable MPVA). Frequency and descriptive analyses were used to examine acceptability and feasibility outcomes. Statistical significance was set at *p* < .05.

## Results

### Participant flow and baseline characteristics

**Figure 1:**
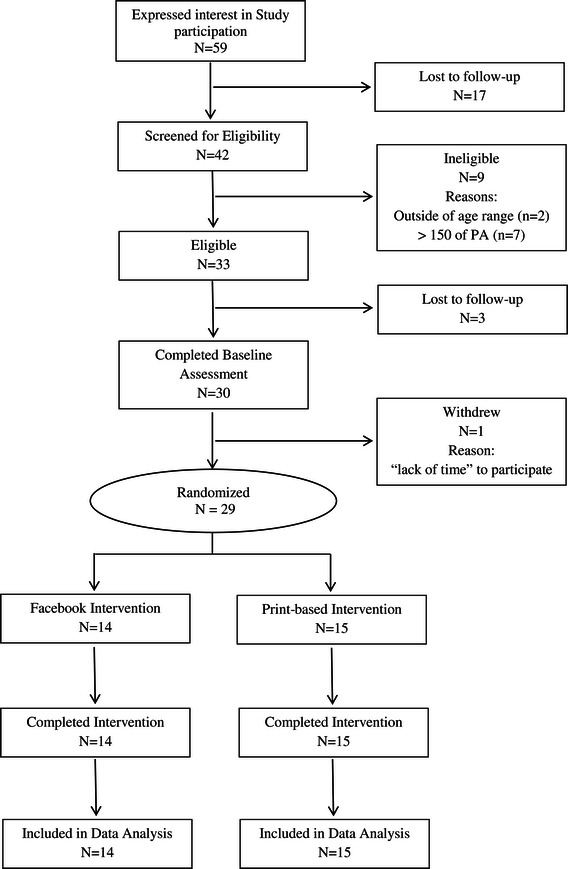
**Consort diagram illustrating participant flow.**

Recruitment efforts resulted in 59 African American women contacting study staff to express interest in the study. Of these 59 women, 42 were screened for eligibility and 33 were eligible to participate. Reasons for ineligibility included performing greater than 150 minutes of moderate-to-vigorous physical activity the week prior to the eligibility screening (n = 7) and being outside the defined age for study participation (n = 2). Among the 33 eligible participants, 30 attended their baseline study assessment and provided full baseline data. However, one participant withdrew from the study prior to being notified of her randomization assignment, resulting in 14 women randomized to the FI group and 15 to the PI group. All randomized participants completed the 8-week study, indicating 100% retention. Figure 1 shows participant flow throughout the 8-week study.

African American women enrolled in the study had a mean age of 35 years (*SD* = 5.0) and the majority were classified as either overweight or obese (*M* BMI = 31.2, *SD* = 7.9). Most were not married (n = 18, 62%) and reported had at least one child living at home having (n = 16, 55%). All women had completed some college coursework with most (n = 26, 89.6%) having a bachelor’s degree or higher. Table 2 illustrates complete baseline characteristics of study participants. No statistical differences (*P* < .05) were observed between the FI and PI groups for demographic variables at baseline.

**Table 2:** **Baseline characteristics of randomized women (N = 29)**

	Total sample (N = 29)	Facebook group (N = 14)	Print group (N = 15)
**Age,** ***M (SD),*** **years**	35.5 (5.0)	35.6 (6.2)	35.3 (3.8)
**BMI,** ***M*** **(** ***SD*** **), kg/m** ^**2**^	31.2 (7.9)	30.0 (7.8)	32.3 (8.3)
**Marital status,** ***N*** **(%)**			
Married	11 (37.9)	4 (28.6)	7 (46.7)
Divorced	4 (13.8)	2 (14.3)	2 (13.3)
Never married	14 (48.3)	8 (57.1)	6 (40.0)
**Number of children living at home,** ***N*** **(%)**			
0	13 (44.8)	8 (57.1)	5 (33.3)
1-2	11 (37.9)	2 (14.2)	9 (60.0)
3 or more	5 (17.2)	4 (28.6)	1 (6.7)
**Highest level of education completed** ***N*** **(%)**			
High School or GED	0 (0)	0 (0)	0 (0)
Some college or technical school	3 (10.3)	2 (14.3)	1 (6.7)
Bachelor’s Degree	10 (34.5)	4 (28.6)	6 (40.0)
Master’s Degree	13 (44.8)	7 (50.0)	6 (40.0)
Doctoral Degree	3 (10.3)	1 (7.1)	2 (13.3)
**Annual household income,** ***N*** **(%)**			
Less than $25,000	3 (10.3)	3 (21.4)	0 (0)
Between $25,000 and $50,000	10 (34.5)	5 (35.7)	5 (33.3)
Between $50,001 and $75,000	5 (17.2)	2 (14.3)	3 (20.0)
Between $75,001 and $100,000	3 (10.3)	0 (0)	3 (20.0)
Over $100,000	8 (27.6)	4 (28.6)	4 (26.7)

***Note:*** No statistical differences in demographic characteristics between groups at baseline. BMI = Body Mass Index. All values rounded to the nearest tenth decimal place; therefore, percentages may not equal 100%.

### Physical activity outcomes

Physical activity outcomes are presented in Table 3. Twenty-eight study participants (97%) provided valid ActiGraph wear time to be included in data analysis (n = 1 FI participant did not provide valid accelerometer wear time at the follow-up assessment). Average wear time for these 28 participants was 786.3 minutes/day at baseline and 840.2 minutes/day at follow-up (i.e., 13.1 and 14.0 hours/day, respectively). There was no difference in overall wear time between the two assessment periods (*P* = .178) or for wear time between study groups at either assessment period (*P* = .653 and *P* = .928, respectively). Preliminary tests also showed that study groups had similar physical activity levels at baseline (*P*’s > .05 for all accelerometer measured outcomes; *P* = .237 for self-reported physical activity).

**Table 3:** **Pre- and post-intervention outcomes for ActiGraph accelerometer and Exercise Vital Sign measured physical activity**

	Baseline			8-weeks				Statistical tests		
Variable	M (SD)	Median (min, max)	25th & 75th percentiles	M (SD)	Median (Range)	25th & 75th Percentiles	Baseline to 8-week change	Unadjusted within group baseline to 8-week change	Adjusted between group difference in baseline to 8-week change	Effect size^d^
							Mean (SD)	*P* ^b^	*P* ^c^	*η* ^2^
**Accelerometer outcomes**										
**Sedentary (0 – 99 ctm** ^**a**^ **)**										
Facebook (n = 13)	7811 (547)	7591 (6598, 8444)	7613, 8229	7740 (545)	7605 (6946, 8592)	7403, 8333	−71 (379)	.542	.026	.251
Print (n = 15)	7797 (549)	7887 (6578, 8498)	7499, 8330	7916 (628)	8046 (6746, 8882)	7576, 8399	118 (753)	.639		
**Light activity (100–759 ctm)**										
Facebook (n = 13)	1218 (345)	1061 (843, 1969)	972, 1410	1313 (319)	1259 (756, 1803)	1008, 1596	95 (210)	.146	.024	.263
Print (n = 15)	1175 (268.)	1193 (834, 1683)	878, 1381	1234 (312)	1225 (743, 2010)	962, 1418	59 (401)	.421		
**Moderate-lifestyle (760–1951 ctm)**										
Facebook (n = 13)	461 (220)	418.0 (198, 824)	216, 713	489 (195)	515 (125, 730)	336, 665	27 (112)	.558	.001	.419
Print (n = 15)	463 (242)	382 (197, 1076)	314, 556	429 (178)	356 (159, 744)	273, 556	−34 (248)	.901		
**Moderate activity (1952–5725 ctm)**										
Facebook (n = 13)	122 (67)	133 (26, 252)	61, 180	113 (63)	109 (24, 220)	59, 164	−9 (78)	.635	.422	.069
Print (n = 15)	132 (99)	120 (10, 332)	57, 201	135 (125)	98 (7, 502)	58, 195	0 (82)	.773		
**Vigorous activity (>5725 ctm)**										
Facebook (n = 13)	2 (5)	0 (0, 19)	0, 0	12 (3)	0 (0, 12)	0, 2	-.31 (7)	.906	n/a	.459
Print (n = 15)	8 (11)	0 (0, 34)	0, 11	4 (10)	0 (0, 38)	0, 3	−4 (12)	.438		
**Total moderate-to-vigorous activity (>1951 ctm)**										
Facebook (n = 13)	123 (69)	137 (23, 252)	61, 180	116 (64)	112 (24, 220)	596, 165	−9 (78)	.735	.327	.089
Print (n = 15)	140 (107)	120 (10, 343)	63, 225	139 (129)	98 (7, 513)	61, 195	−1 (87)	.890		
**Moderate-to-vigorous activity in 10 minutes bouts or greater (>1951 ctm)**										
Facebook (n = 13)	22 (27)	10 (0, 85)	0, 40	26 (35)	11 (0, 106)	0, 41	4 (45)	.910	.637	.036
Print (n = 15)	33 (44)	12 (0, 121)	0, 40	36 (72)	0 (0, 258)	0, 68	3 (57)	.831		
**Exercise vital sign** ^**c**^ **(minutes/week)**										
Facebook (n = 14)	91 (120)	60 (0, 450)	18, 98	153 (120)	110 (20, 450)	75, 217	62 (169)	.025	.015	.483
Print (n = 15)	101 (90)	90 (10, 360)	30, 120	108 (1445)	90 (0, 600)	40, 100	7 (194)	.801		

Facebook = Facebook and text message intervention (n = 13 for accelerometer outcomes because one participant did not provide valid wear time for analysis at 8-weeks); Print = Print-based intervention (n = 15 for accelerometer outcomes).

All reported physical activity values are in in minutes/week and are rounded to the nearest integer.

^a^ctm = counts per minute.

^b^Wilcoxon signed rank *P* for within group baseline to 8 week change.

^c^Between group differences in baseline to 8-week physical activity changes calculated using ANCOVAs and adjusted for baseline physical activity levels.

^d^Eta squared effect size estimates for ANCOVA models controlling from baseline physical activity levels.

Results from Wilcoxon signed rank tests used to examine within-group physical activity changes showed no significant baseline to 8-week changes for any of the accelerometer measured physical activity outcomes for either intervention group (*P*’s > .05). ANCOVA models (controlling for baseline physical activity levels) showed significant between-group differences for changes in sedentary behavior (*P* = .026), light-intensity physical activity (*P* = .024), and moderate-lifestyle intensity physical activity *(P* < .001). Specifically, women in the FI decreased sedentary behavior by 71 minutes/week in comparison to women in the PI who increased sedentary behavior by 118 minute/week (*P* = .026 difference between groups, *η*^*2*^ = .251). Increases in light intensity physical activity were observed in both study groups (FI = 95 minutes/week vs. PI = 59 minutes/week), with the FI demonstrating significantly greater increase than the PI (*P* = .024 for difference between groups, *η*^*2*^ = .263). Similarly, the FI increased moderate-lifestyle intensity physical activity by 27 minutes/week while women the PI decreased moderate-lifestyle physical activity by 35 minutes/week (*P* < .001 for difference between groups, *η*^*2*^ = .419). No other between groups differences were observed for accelerometer measured outcomes, including: moderate-intensity physical activity (*P* = .422), moderate-to-vigorous physical activity (*P* = .317) or moderate-to-vigorous intensity physical activity performed in bouts of 10-minutes or greater (*P* = .637).

Self-reported physical activity using the Exercise Vital Sign Questionnaire showed that women in the FI significantly increased moderate-to-vigorous physical activity by a median 50 minutes of from baseline to 8 weeks (*P* = .025) while self-reported moderate-to-vigorous physical activity among PI participants remained relatively stable (*P* = .801). Subsequent analysis using ANCOVA (controlling for baseline physical activity) showed that self-reported increases in physical activity among FI participants were statistically greater than changes reported by women in the PI (*P* = .015, *η*^*2*^ = .483).

**Figure 2:**
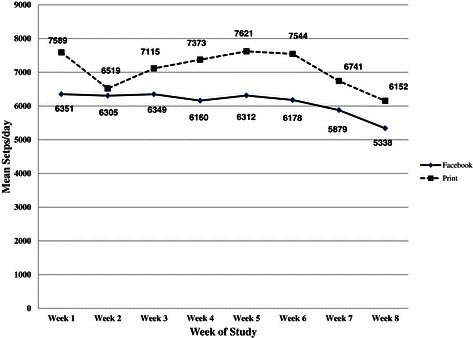
**Mean weekly pedometer-measured steps per of day according to intervention group.**

Although not a formal outcome of the study, we also examined the weekly average number of steps per day self-reported by participants over the duration of the 8-week study. As illustrated in Figure 2, PI participants, on average, performed a higher number of steps throughout the 8-week study. The average number of steps per day for both groups remained relatively stable during first six weeks of the study (with the exception of the week 2 decline in the PI) and subsequently declined during the last two weeks of the intervention period. Wilcoxon singed rank tests examining within-group changes in steps/day from week 1 to week 8 showed a significant decrease in steps/day among the FI participants (*P* = .016) and no change among women in the PI (*P* = .152). However, when outcomes were evaluated using ANCOVA (controlling for week 1 daily steps), results showed no between-group difference in changes in steps/day between week 1 and week 8 of the study (*P* = .100).

### Social Cognitive Theory and BMI outcomes

Table 4 illustrates the BMI and Social Cognitive Theory outcomes for both study groups. The PI and FI groups had no statistically significant differences in BMI or Social Cognitive Theory variables at baseline. At the end of the 8-week study, participants receiving the FI reported significant pre-post intervention enhancements in self-regulation for physical activity (*P* < .001) and social support from family for physical activity (*P* = .030). Conversely, the only significant Social Cognitive Theory enhancement reported by the PI group was for self-regulation for physical activity (*P* < .001). Subsequent ANCOVA analyses showed that enhancements in self-regulation for physical activity and social support from family for physical activity among FI participants were significantly greater than enhancements reported by PI participants (*P* < .001 *η*^*2*^ = .575; *P* = .044, *η*^*2*^ = .262; respectively). Unexpectedly, a significant between group difference (*P* = .001, *η*^*2*^ = .392) for changes in outcome expectations for physical activity that favored the PI also emerged. No pre-post intervention changes in exercise self-efficacy or social support from friends were reported by either intervention group. BMI among all study participants remained stable over the duration of the 8-week study (*P* = .844).

**Table 4:** **BMI and Social Cognitive Theory outcomes**

		Baseline		8-Weeks					
Variable	Range^a^	M (SD)	Median	M (SD)	Median	Baseline to 8-week change	Unadjusted baseline to 8-week change	Adjusted between group difference in baseline to 8 week change	Effect size^d^
						Mean (SD)	*P* ^b^	*P* ^c^	*η* ^2^
**BMI (weight [kg]/height [m** ^**2**^ **])**									
Facebook (n = 14)	19.90-55.00	30.01 (7.58)	28.05	29.95 (7.31)	27.60	-.06 (.82)	.866	.502	.082
Print (n = 15)		32.29 (8.34)	30.20	32.62 (8.21)	30.90	.32 (1.76)	.718		
**Outcome expectations**									
Facebook (n = 14)	1-5	4.31 (0.40)	4.39	4.46 (.36)	4.44	.15 (.32)	.114	.001	.392
Print (n = 15)		3.94 (.55)	4.00	4.200 (4.29)	4.11	.26 (.49)	.081		
**Self-regulation**									
Facebook (n = 14)	1-5	1.69 (.56)	1.55	3.21 (.47)	3.22	1.51 (.63)	<.001	<.001	.575
Print (n = 15)		1.76 (.38)	1.80	2.73 (.45)	2.50	.96 (.69)	<.001		
**Self-efficacy**									
Facebook (n = 14)	1-5	3.58 (.61)	3.57	3.73 (.72)	3.57	1.14 (.49)	.385	.280	.092
Print (n = 15)		3.53 (.63)	3.38	3.76 (.74)	3.64	.23 (.71)	.246		
**Social support from friends**									
Facebook (n = 14)	8-40	18.62 (7.81)	20.00	23.85 (7.66)	26.66	5.23 (9.77)	.099	.366	.080
Print (n = 15)		17.57 (8.94)	15.50	19.57 (11.29)	16.00	3.47 (8.90)	.403		
**Social support from family**									
Facebook (n = 14)	10-50	20.64 (4.60)	20.00	27.00 (8.29)	24.50	3.36 (9.44)	.030	.044	.262
Print (n = 15)		17.78 (5.69)	16.00	20.38 (9.59)	17.00	2.71 (10.23)	.671		

^a^BMI range is the observed minimum and maximum for entire sample; questionnaire range scores are the potential range for each respective measure (the higher the score the more favorable the outcome).

^b^Wilcoxon signed rank for baseline to 8 week change.

^c^Mann–Whitney p-value examining the difference in baseline to 8-week change values between groups. BMI = Body Mass Index.

^d^Eta squared effect size estimates for ANCOVA models controlling from baseline values.

### Acceptability and feasibility

Favorable outcomes were reported for acceptability and feasibility of the Facebook and text message approach to promote physical activity. One hundred percent (N = 14) of participants in the FI group reported gaining physical activity knowledge from culturally-adapted physical activity promotion materials, compared to only 53% (n = 8) of participants in the PI group. Similarly, 94% (n = 13) of FI participants reported that they were “motivated” or “very motivated” to continue being physically active after the completion of study (n = 1 was “somewhat motivated”), in contrast to only 7% (n = 1) of women in the PI (n = 5 [33.3%] indicated they were “not motivated” and n = 9 (60%) reported they were “somewhat motivated”). In reference to the physical activity promotion text messages sent to FI participants, 93% (n = 13) reported gaining physical activity-related knowledge from the messages and 79% (n = 11) indicated they were “helpful” to “very helpful” for promoting physical activity (n = 3 reported them as “somewhat helpful”).

Seventy-nine percent (n = 11) of FI participants reported the physical activity promotion materials were “helpful” to “very helpful” for promoting physical activity (3 participants [21.4%] reported them as “somewhat helpful”), 86% (n = 12) reported that were “satisfied” or “very satisfied” with the program (n = 2 were “somewhat satisfied”), and all (100%) reported they would recommend the culturally-relevant Facebook and text-message delivered physical activity program to a friend. In comparison, only 26.6% (n = 4) of participants in the print group reported their physical activity promotion materials as “helpful” for promoting physical activity (n = 3 [20%] reported them as “not helpful at all”, n = 8 [53.3%] reported them “somewhat helpful”, and n = 0 reported them as “very helpful”). Moreover, only 47% (n = 7) of the print-based group participants were “satisfied” or “very satisfied” with the program (n = 7 were “somewhat satisfied” and n = 1 was “not satisfied at all”) and 87% (n = 13) indicated that they would recommend the print-based physical activity promotion program to a friend.

Written qualitative feedback provided by participants on the post-intervention satisfaction survey also supported the culturally-relevant physical activity program delivered via Facebook and text messaging. Example quotes from participants regarding the physical activity program included: “It [the study] provided a sense of accountability and it was good to hear what other participants were saying [on the Facebook wall].”, “The program was good and I think it will be helpful to others.”, “The study made me think about being more physically activity and I will incorporate scheduling exercise on my calendar in the future.”, “Loved the information uploaded on the Facebook page regarding physical activity.”, and “Repeat the study, it is very much needed in my community”.

### Fidelity of intervention delivery and participant engagement

All eight of the weekly Facebook posts and discussion topics were successfully delivered on the study Facebook wall as scheduled. Table 5 illustrates the number of participants who viewed and commented on the weekly physical activity promotion posts and group discussion topics. Analytic tracking software provided by Facebook indicated that all study participants (100%) viewed the weekly physical activity promotion posts and group discussion topics during the first half of the study (i.e., weeks 1–4); however, during the latter half of the study, the number of participants who viewed the weekly physical activity promotional posts and group discussion topics declined slightly (i.e. 64-86%). Additionally, half (n = 7) of the FI participants viewed all the physical activity promotion materials posted to the group wall and the median number of participant comments for each weekly group discussion topic was 5.5 (*M* = 5.75).

**Table 5:** **Number of participants who viewed and commented on each Facebook post**

Week	Weekly physical activity promotion topic	Number (%) of participants who viewed the weekly physical activity promotion post	Number of comments on the weekly physical activity promotion post	Number of participants commenting on the weekly physical activity promotion post	Number (%) of participants who viewed the weekly group discussion topic	Number of comments on the group discussion topic	Number of participants commenting on the group discussion topic
1	Overview of National physical activity Recommendations, Health benefits of physical activity, and physical activity statistics among AA women.	14 (100)	8	4	14 (100)	4	3
2	Developing a physical activity plan that works for you.	14 (100)	3	3	14 (100)	9	6
3	Barriers to physical activity among AA women and strategies to overcoming barriers.	14 (100)	5	4	14 (100)	9	5
4	Developing a Social Support network to promote physical activity.	14 (100)	0	0	14 (100)	9	9
5	Strategies for incorporating short bouts of physical activity into your daily routine to achieve national physical activity recommendations.	12 (86)	2	2	10 (67)	1	1
6	Testimonials from AA women on how they successfully incorporate physical activity into their daily lives.	10 (67)	1	1	12 (86)	5	5
7	*Revisited:* National physical activity recommendations, Barriers to physical activity among AA women and strategies to overcoming barriers, and strategies to incorporate more physical activity into your life.	11 (79)	7	4	11 (79)	6	6
8	Strategies for maintaining a physically active lifestyle after the intervention.	9 (64)	4	4	9 (64)	4	4

## Discussion

The purpose of the current pilot study was to evaluate the potential use of Facebook and text-messaging to deliver a culturally-relevant physical activity promotion intervention to African American women. Results showed several promising outcomes, including decreased sedentary behavior, increased light and moderate-lifestyle intensity physical activity, and enhanced self-regulation and social support from family for physical activity. Moreover, favorable findings regarding acceptability and feasibility lend further support for the approach to promote physical activity among African American women. Results provide important preliminary insight regarding the use of existing social media infrastructures and mobile phone technology to deliver culturally-relevant physical activity interventions to African American women.

While participants in both intervention groups failed to demonstrate significant increases in accelerometer measured moderate-to-vigorous intensity physical activity, FI participants decreased their sedentary time and increased time spent in light- and moderate-lifestyle physical activity over the 8-week study when compared to PI participants. Moreover, the between group effect sizes for these physical activity outcomes were especially promising for the FI (i.e. *η*^*2*^ = .251 for sedentary behavior, *η*^*2*^ = .263 for light physical activity, and *η*^*2*^ = .419 for moderate-lifestyle physical activity) as they were substantially larger than the mean effect size (*d* = .19) reported in a recent meta-analysis of physical activity interventions for healthy adults [51]. The reduction in sedentary behavior and increases in lower-intensity physical activity are particularly relevant considering FI participants self-reported increases in moderate-to-vigorous intensity physical activity (as measured by the Exercise Vital Sign Questionnaire). These findings suggest that FI participants actually increased their overall physical activity; however, contrary to their perceptions, the intensity of the physical activity performed was lower than the threshold of moderate-intensity according to accelerometers. This outcome suggests the need for inclusion of an objective measure of physical activity and an intervention component to show participants how it “feels” to engage in moderate-intensity physical activity. Example strategies may include having participants walk at a moderate-intensity with study staff during their baseline assessment or engage in a brief moderate-intensity aerobic physical activity session.

The seasonal time period in which the study was conducted may have also played a role in the null accelerometer measured moderate-to-vigorous intensity physical activity outcomes, as well as the slight decline in daily pedometer-measured steps performed during the last two weeks of the 8-week study. The study was conducted in Phoenix, Arizona during March – June (i.e., late spring/early summer months), a period when daily high temperatures increased dramatically. The average daily high temperature during the two-week period in which participants wore accelerometers at baseline was 82 degrees Fahrenheit. In comparison, the average daily high temperature during the follow-up data collection period was 103 digress Fahrenheit. This approximate 21 degree rise in temperature may have influenced physical activity levels among participants as temperature during the follow-up assessment period may have been too hot for most women to perform outside activities or to walk places. Temperature and heat-related concerns may have been further accentuated in our sample as many African American women identify sweating and sun exposure as prominent barriers to physical activity [50,52-55].

Both intervention groups reported significant increases in self-regulation for physical activity over the 8-week study; however, the FI group reported significantly greater enhancements in self-regulation than the PI group (*P* < .001). We attribute the enhancements in self-regulation in both study groups to the pedometer and self-monitoring components of the intervention. Further, the finding that FI participants reported significantly greater increases in self-regulation than the PI group suggest that the FI intervention components were more effective in promoting self-regulation for physical activity; however, due to design limitations, we are unable to determine the specific influence each of the FI intervention components (i.e., the culturally-relevant physical activity promotion materials or adaptive pedometer-based self-monitoring approach) had on this Social Cognitive Theory construct.

We also observed a significant increase in social support from family over the 8-week study among FI participants. This finding was encouraging and suggests that participants may have used some of the social support strategies presented FI materials foster support for physical activity among family members (i.e., Week 4 included a specific topic on how to facilitate social support for physical activity from family members). Conversely, the lack of pre-post intervention changes in exercise self-efficacy for both intervention groups was unexpected. However, comparable null outcomes have been reported in previous website-based interventions promoting physical activity among African American women [8,9]. Potential explanations for the lack of change in this variable may be associated with the relatively high scores reported by participants at baseline, which limited room for improvement, or that as participants began to increase their physical activity levels they realized that physical activity was more difficult than they anticipated. Another unanticipated outcome was that participants in the PI reported greater enhancements in outcome expectations for physical activity than participants in the FI. This perhaps speaks to the high quality of the content in the print-materials the PI received and indicates the need for the FI materials to be refined to further address this Social Cognitive Theory construct.

Findings regarding the acceptability and feasibility of the Facebook and text-message approach to promote physical activity were generally positive. Self-reported satisfaction and treatment acceptance of the FI was high, with all reporting that they gained physical activity-related knowledge from the weekly Facebook posts and all indicating they would recommend the study to a friend. Moreover, an overwhelmingly majority of FI participants (i.e. 93%, n = 13) reported they were “motivated” or “very motivated” to continue to be physically active after the end of the study. We suggest that the favorable acceptance among FI participants was associated with the culturally-relevant approach used to promote physical activity, as previous research suggest culturally-relevant behavior change interventions are advantageous in increasing the receptivity and perceived salience of the targeted behavior [30,56]. Further, the use of pre-existing communication channels (i.e. Facebook and text messages) to deliver the physical activity intervention likely contributed to the high participant acceptance, as it minimized participant burden associated with accessing/obtaining the intervention materials. An interesting outcome was that despite only 53% of PI participants indicating they gained knowledge from the print-based program and less than half (47%) reported being “satisfied” or “very satisfied” with the program, the majority (87%, n = 13) indicated they would recommend the intervention to a friend. We hypothesize that this discrepancy may have been influenced by social desirability as PI participants likely wanted to provide favorable information regarding the intervention to study staff.

Treatment receipt also supported the feasibility of using Facebook and text messages to deliver a culturally-relevant physical activity program to African American women. However, despite high fidelity of intervention delivery, a disappointing outcome was the decline in participant receipt of intervention materials during the second half of the study (i.e., 100% for the first 4 weeks of the study vs. 64-86% during week 5–8). Participant interaction on the group Facebook wall was also lower than expected with the average number of 5.5 participant comments on the weekly discussion topic post. We speculate that modest participant engagement on the Facebook wall may have contributed the non-significant change in social support for physical activity from friends, as we anticipated this component would penetrate and enhance this Social Cognitive Theory construct. However, since the social support survey did not differentiate between study “Facebook friends” and friends outside of the study, the exact role of participant engagement on the Facebook wall cannot be ascertained. These results indicate the need for additional strategies to promote and maintain participant engagement with study activities as we anticipated participants would be more interactive on the group Facebook page. Examples strategies for future research may include adding an in-person component at the beginning of the study for participants to interact and get to know each other prior to expecting them to interact on the Facebook page or modifying the physical activity promotion material to be more visually appealing and/or more interactive by incorporating short video and/or audio clips to complement the print-based materials.

Several study limitations must be noted. Due to the small sample size, we were likely underpowered to detect significant pre-post intervention changes in various physical activity, anthropometric, and psychosocial outcomes. However, the primary focus of the study was to evaluate the acceptability and feasibility of using Facebook and text-messages to deliver a culturally-relevant physical activity program—for which the sample size is appropriate. Additionally, the sample was comprised of a convenience sample of highly educated African American women (i.e., all had a high school degree and most had a college degree) living in the Phoenix metropolitan area. Therefore, the findings may not be generalizable to African American women of different socioeconomic backgrounds or those residing in other geographical regions. Similarly, Facebook and text message materials were written at a 10th grade reading level or lower. In future research, we plan to reduce the reading level of study materials (i.e., to a 5th grade reading level) to enhance readability and improve the potential for broader dissemination of the program. We also did not assess frequency of Facebook use or level of Facebook knowledge/skill during recruitment efforts. Including such measures will be considered in future research as more frequent Facebook use and higher knowledge on how to navigate the Facebook website may have influenced study outcomes.

Another limitation was that text messages were not individually tailored according to participant-specific characteristics. All participants received the exact same text messages over the 8-week study. Given the content of the some of the text messages may not have been relevant to all women enrolled in the study (e.g., one text message focused on overcoming childcare barriers to physical activity and only half of FI participants had children), individually tailoring messages to address participant-specific characteristics and barriers to physical activity will be included in future studies. The study also included an active control group that received a non-tailored physical activity intervention. Inclusion of a passive control group would have been beneficial to compare changes in physical activity levels among women not receiving physical activity promotion materials over the 8-week intervention period. However, inclusion of a 3rd group was not possible due to funding limitations. We also note that two different pedometer-based self-monitoring approaches were used in the intervention groups; limiting our ability to determine the exact influence each of these strategies had on study outcomes. The multi-component nature of the Facebook and text message study arm also limits our ability to determine which aspects of the study were beneficial to behavior change (e.g., delivery channel, cultural adaption, combination of both). Future research and larger studies are needed to explore the influence that each of these components had on the physical activity and associated psychosocial outcomes of the study. Lastly, the study focused on relatively short-term physical activity and psychosocial outcomes following the 8-week intervention period. Future studies are needed to evaluate the longer-term behavioral and psychological outcomes (i.e., 6 and 12 months post-intervention) following the intervention period as long-term adoption of healthy behaviors are imperative to chronic disease prevention.

Despite these limitations, the current study has several strengths. A limited number of studies have used a social media-based approach to deliver a physical activity promotion intervention to African American women. Given the favorable findings, future research is warranted to further examine the use of these technology-based platforms to promote physical activity in African American women. Second, we used a culturally-relevant approach when developing the physical activity promotion materials that addressed prevalent sociocultural norms, traditions, and barriers associated with physical activity among African American women. Culturally-tailoring intervention activities have the potential to increase the acceptability and uptake of a behavioral interventions, which can ultimately lead to positive changes in the targeted and behavioral outcome of interest [30,57,58]. Moreover, we grounded study activities in behavioral health theory (i.e., Social Cognitive Theory), which can also increase the potential efficacy of the physical activity program [59]. Use of accelerometers to measure physical activity was a strength. Accelerometers are advantageous because they are not subject common issues associated with questionnaire or survey assessment such as recall bias, same source bias, or the necessity for high literacy and/or numeracy levels to comprehend questions and estimate time spend in physical activity. The high adherence of accelerometer wear among study participants (i.e. 97%) also strengthened the credibility of the physical activity outcomes. A final strength was the 100% retention of randomized participants, especially given the moderate-to-high attrition commonly reported by physical activity studies with African American women [60] and studies using technology-based mediums to promote physical activity [13-15].

## Conclusions

The current study provides important insight regarding the acceptability and feasibility of using social media and text messages to promote physical activity in African American women. Overall, study findings suggest that that culturally-relevant, Facebook and text message delivered physical activity program was associated with a reduced sedentary behavior, increased light and moderate-lifestyle physical activity, and was well-received among study participants. The lack of significant increases in moderate-to-vigorous physical activity calls for future research to further explore the use of these technologies to successfully promote physical activity. Similarly, due to the limited amount of research on this topic, there is the potential for additional qualitative research to explore the role technology-based platforms can play in promoting physical activity among African American women. As time and technology continue to progress at a rapid pace, researchers developing and implementing physical activity programs for African American women should stay abreast of emerging technology trends in order to adapt the delivery methods of their culturally-relevant behavior change programs.

## Abbreviations

FI: Facebook and text message intervention
PI: Print-based intervention
BMI: Body mass index
